# New models for prediction of micronuclei formation in nuclear medicine department workers

**DOI:** 10.1186/s12995-015-0066-5

**Published:** 2015-07-25

**Authors:** Sanja Terzic, Aleksandar Milovanovic, Jelena Dotlic, Boban Rakic, Milan Terzic

**Affiliations:** Occupational Health Department, General Health Center “Savski Venac”, Pasterova 1, Belgrade, Serbia; Institute of Occupational Medicine and Radiological Protection, Deligradska 29, Belgrade, Serbia; School of Medicine, University of Belgrade, Dr Subotica 8, Belgrade, Serbia; Clinic of Obstetrics and Gynecology, Clinical Center of Serbia, Dr Koste Todorovica 26, Belgrade, Serbia

**Keywords:** Professional exposure to radiation, Nuclear medicine, Micronucleus, Chromosomal damage

## Abstract

**Background:**

Ionizing radiation causes detrimental health effects such as cancer and genetic damage. The study aim was to determine predictors for micronuclei (MN) occurrence and frequency in peripheral blood lymphocytes of health workers professionally exposed to radiation.

**Methods:**

Health workers, age matched, selected for the study on regular check-ups, were divided according to the radiation exposure. The exposed group involved nuclear medicine department employees (54) and the control group comprised workers from other departments (36). Data about workers characteristics and habits, received annual doses (AD), total years of service (TYS) and exposed years of service (EYS) were taken from each subject. Blood samples were taken and micronuclei (MN) number in peripheral blood lymphocytes was calculated using CBMN assay according to standard protocols.

**Results:**

Most workers were female, technicians, with mean age of 45.67 years and EYS about 15 years. Health workers exposed to radiation had significantly more MN than controls (*p* = 0.001). Female gender, older age, higher received annual doses, longer EYS and TYS increased the MN number. Technicians and laboratory workers have higher risk for MN occurrence. Significant predictors of MN formation according to constructed model were workers age, sex, AD and EYS. One EYS year increases MN frequency 1.017 times, while receiving 0.1 mSy raises MN frequency by 26 %. EYS accurately predicts 86.30 % of MN frequencies and AD 64.60 %.

**Conclusions:**

The model, developed for the first time in this study, showed that received annual doses and duration of exposure to radiation can be used for prediction of MN numbers.

## Background

The risk related to low doses of radiation is primarily due to stochastic effects [1]. The main target of ionizing radiation is the DNA molecule. Adverse biological effects of ionizing radiation are gene mutations, chromosomal rearrangement, cellular transformation, micronucleus [MN] formation, apoptosis, and radiation carcinogenesis [2, 3]. Medical personnel represent the group that is most consistently exposed to low doses of ionizing radiation, due to the widespread use of ionizing radiation as a diagnostic and therapeutic tool [4]. Various studies have been published showing increased frequencies of chromosomal aberrations in health workers occupationally exposed to low levels of radiation [5–7]. Testing for occurrence and frequency of micronuclei in peripheral blood lymphocytes is usually performed to evaluate cytogenetic damage in populations occupationally exposed to ionizing radiation [1, 4].

The aim of this study was to determine potential predictors for development of cell damage, i.e., the occurrence and frequency of micronuclei in peripheral blood lymphocytes of health workers professionally exposed to radiation.

## Methods

We analyzed health status, characteristics and habits, as well as specific working places of health care workers professionally exposed to radiation. The study was approved by the Ethical Committee and the written consent for participation in the study was obtained from each subject.

According to the radiation exposure, all examined health workers were divided into the exposed and the control group. The exposed group consisted of all employees who worked with open sources of ionizing radiation at the Institute of Nuclear Medicine, Clinical Center of Serbia, under the supervision of the Radiological Protection Institute of Occupational Health of Serbia “Dr. Dragomir Karajovic”. Workers from the exposed and control groups were matched by age and comparable according to other characteristics, in order to reduce probable confounding bias. The control group comprised health workers from Clinical Center of Serbia, who had regular check-ups in the Institute of Occupational Health of Serbia “Dr. Dragomir Karajovic” and were not in contact with radiation.

Detailed anamnesis, with questions about sex, age, use of nicotine, total years of service (TYS) and occupation (doctor, technician, laboratory worker and engineer), was taken from each subject. For workers exposed to radiation we also determined exposed years of service (EYS) and received annual doses (AD), based on data from their personal dosimeters that were taken from medical records of periodic examinations at the Institute of Occupational Health of Serbia “Dr. Dragomir Karajovic”.

Samples of 5 ml of whole blood were taken from all subjects by heparinized vacutainer tubes, and kept at room temperature for less than 6 h. Using CBMN assay (Cytokinesis-block MicroNucleus Assay) cells were cultivated according to standard protocols [8]. The blood sample was mixed with standard serum based culture medium – RPMI 1640 medium supplemented with calf serum, l-glutamine and penicillin as well as 1.5 % phytohaemagglutinin (PHA) that is added in order to stimulate lymphocytes division [8]. The mixture is cultivated at 37 °C in a 5 % CO_2_ during 72 h. Cytochalasin B was added 44 h after culture initiation. Finally, the preparation was centrifuged to collect cells [8]. Drop of this mixture was placed on the plate, fixed and dried. Cell staining was done in the traditional manner (Giemsa). Obtained preparations were analyzed under the light microscope 400× magnification (Olympus BX-51, magnification 1000 h). In each sample, a single operator examined 1000 binuclear cells with a well-preserved cytoplasm. The presence of micronuclei was registered and their number determined per 1000 binuclear cells. This analysis was performed according to the standard criteria established in the HUMN project (Human MicroNucleus Project) [9].

Obtained data were statistically analyzed using Kolmogorov-Smirnov test, *χ*2 test, Student’s *t*-test, ANOVA to test significance of differences in patient frequencies regarding categories of evaluated parameters as well as between groups (exposed and unexposed). Spearman’s correlation was used to assess the influence of investigated parameters on MN formations. We applied Receiver Operator Curve (ROC) analysis to determine potential predictors of radiation damage to cells. Poisson’s regression (generalized linear modeling) was used to create model of relationship between tested parameters and MN frequency. Data were analyzed using the Statistical Package for the Social Sciences (SPSS) software (Advanced Statistics, version 17.0, SPSS Inc. - Chicago, IL, USA).

## Results

Study involved 90 workers divided in two groups: exposed group with 54 persons professionally exposed to radiation and control group consisting of 36 workers that were not in contact with radiation.

The investigated workers were 45.67 years old on average (Table 1). The majority of workers were female and non-smokers, in the overall population as well as in both groups (Table 2). Furthermore, in both groups, most of the workers were technicians (Table 2). Average TYS was 18.8 years, while mean EYS, assessed only in exposed workers, was 15 years (Table 1). Technicians and laboratory workers cumulated significantly more EYS than doctors and engineers (*p* = 0.004).

**Table 1:** Descriptive parameters of examined health workers in exposed and control groups

	Parameters	Overall population	Exposed group	Control group
AGE	Mean	45.67	45.67	45.67
	Standard deviation	10.48	10.48	10.48
	Minimum	28.00	28.00	28.00
	Maximum	64.00	64.00	64.00
TYS	Mean	18.80	19.31	18.03
	Standard deviation	10.39	10.83	9.78
	Minimum	1.00	1.00	3.00
	Maximum	35.00	35.00	34.00
EYS	Mean	14.98	14.98	0.00
	Standard deviation	10.03	10.03	0.00
	Minimum	1.00	1.00	0.00
	Maximum	35.00	35.00	0.00
MN per 1000 binuclear cells	Mean	13.28	18.39	5.61
	Standard deviation	10.74	10.81	3.92
	Minimum	0.00	3.00	0.00
	Maximum	59.00	59.00	14.00

*MN* micronucleus, *TYS* total years of service, *EYS* exposed years of service

**Table 2:** Frequency of examined workers in different categories of examined parameters in exposed and control groups

Patient characteristics		Overall population		Exposed group		Control group	
		No	%	No	%	No	%
Age categories - decades	3 - 20 – 29 y	8	8.90	4	7,41	4	11,11
	4 - 30 – 39 y	20	22.20	12	22,22	8	22,22
	5 - 40 – 49 y	21	23.30	16	29,63	5	13,89
	6 - 50 – 59 y	37	41.10	20	37,04	17	47,22
	7 - 60 – 69 y	4	4.40	2	3,70	2	5,55
	*χ* ^2^	25.074		16.872		21.095	
	p	0.001		0.001		0.001	
Sex	Male	26	28.90	10	18,50	16	44,44
	Female	64	71.10	44	81,50	20	55.56
	*χ* ^2^	16.044		21.407		0.444	
	p	0.001		0.001		0.505	
Occupation	Technician	40	44.40	22	40,70	18	50,00
	Lab. worker	15	16.70	8	14,80	7	19,44
	Doctor	29	32.20	18	33,30	11	30,56
	Engineer	6	6.70	6	11,10	0	0
	*χ* ^2^	30.089		13.259		5.467	
	p	0.001		0.004		0.046	
MN categories (intervals of 10) per 1000 binuclear cells	1 cat: 1 – 9	43	47.80	13	24,07	30	83,33
	2 cat: 10 – 19	14	15.60	8	14,81	6	16,67
	3 cat: 20 – 29	22	24.40	22	40,74	0	0
	4 cat: 30 – 39	10	11.10	10	18,52	0	0
	5 cat: 40 – 49	0	0	0	0	0	0
	6 cat: 50 – 59	1	1.10	1	1,85	0	0
	*χ* ^2^	29.444		12.602		16.000	
	p	0.001		0.001		0.001	
Smoking	Yes	27	30.00	17	31,50	10	27,78
	No	63	70.00	37	68,50	26	72,22
	*χ* ^2^	14.400		7.407		7.111	
	p	0.001		0.006		0.008	

*Lab* laboratory, *MN* micronucleus, C*at* category; *Y* years

Investigated nuclear medicine department workers received on average 1.37 +/− 0.29 mSy (min = 0.74 mSy; max = 3.71 mSy) of radiation during one year period. The mean received annual doses for doctors was 1.02 +/− 0.33, engineers 0.86 +/− 0.42, technicians 1.95 +/− 0.67, while for laboratory workers AD were 1.69 +/− 0.51. Received annual doses of all workers were in the referral range. There were no significant differences (*p* > 0.05) between received AD between all four assessed occupations, but technicians and laboratory workers evaluated together, received significantly higher AD than doctors and engineers (*p* = 0.039).

Investigating the number of MN in the overall population we found that the mean MN number was 13.28 per 1000 binuclear cells (Table 1). The majority of workers in the overall population, as well as in the exposed and control group had 1 to 9 MN per 1000 binuclear cells. Moreover, in the control group all workers had less than 20 MN per 1000 binuclear cells (Table 2).

In order to have more bias control of the data we tested differences in distribution of male and female workers in different categories of age, occupation and MN number. As there were no significant differences regarding, we eliminated sex skewing the data as a problem (Table 3).

**Table 3:** Distribution of male and female workers in different categories of age, occupation and MN number per 1000 binuclear cells

Parameters		Overall population		Exposed group		Control group	
		Male	Female	Male	Female	Male	Female
Age categories - decades	3 - 20 – 29 y	1	7	1	4	0	3
	4 - 30 – 39 y	10	10	4	6	6	4
	5 - 40 – 49 y	8	13	2	10	6	3
	6 - 50 – 59 y	6	31	3	21	3	10
	7 - 60 – 69 y	1	3	0	3	1	0
	*χ* ^2^	9.173		4.351		8.834	
	p	0.057		0.361		0.065	
Occupation	Technician	9	31	2	20	7	11
	Lab. worker	6	9	2	6	4	3
	Doctor	9	20	4	14	5	6
	Engineer	2	4	2	4	0	0
	*χ* ^2^	1.819		2.555		0.687	
	p	0.611		0.465		0.709	
MN categories (intervals of 10) per 1000 binuclear cells	1 cat: 1 – 9	11	13	5	8	6	5
	2 cat: 10 – 19	4	11	0	8	4	3
	3 cat: 20 – 29	8	25	4	18	4	7
	4 cat: 30 – 39	3	13	1	9	2	4
	5 cat: 40 – 49	0	2	0	1	0	1
	*χ* ^2^	5.350		5.955		2.303	
	p	0.253		0.203		0.680	

*Lab* laboratory, *MN* micronucleus, *Cat* category, *Y* years

There was no statistically significant differences between groups (exposed and control) concerning the TYS, occupation, nicotine use and age decades of workers (*p* > 0.05). On the other hand, there was a significant difference in the number of micronuclei observed between the groups (exposed and control). Significantly more micronuclei have been observed in samples of the exposed workers compared to controls (*p* = 0.001).

There were significant differences in MN number per 1000 binuclear cells in the overall population regarding workers sex and age, AD, TYS and EYS. Women (*p* = 0.012) and older workers (*p* = 0.001) had more MN per 1000 binuclear cells. Health workers who received higher annual doses had more MN per 1000 binuclear cells (*p* = 0.026). Furthermore, workers who with longer EYS (*p* = 0.001) and TYS (*p* = 0.001) had more MN per 1000 binuclear cells. There were no significant differences in the number of MN among the various occupations of the examined health workers from both groups (*p* > 0.05).

When we examined only those health workers who were professionally exposed to radiation, the frequency of registered MN per 1000 binuclear cells was not significantly different regarding sex (*p* = 0.065), nicotine use (*p* = 0.086) and age decades (*p* = 0.370). The difference in MN was also not significant between all four occupation categories (*p* = 0.931), but technicians and laboratory workers together had significantly more MN per 1000 binuclear cells than doctors and engineers combined (*p* = 0.010).

Female sex was significantly correlated with EYS and MN number, while male sex was in correlation with nicotine use. Age was in positive correlation with AD, EYS, TYS and number of MN per 1000 binuclear cells. EYS was significantly and positively correlated with, AD, TYS and MN number. TYS was significantly and positively correlated with number of MN per 1000 binuclear cells, annual doses, nicotine use and age. Annual doses were not significantly correlated with nicotine use and workers’ sex (Table 4).

**Table 4:** Correlations of investigated parameters

Parameters		Sex	Age	Occup.	EYS	TYS	MN No	Nicotine use	Group exp/cont
Sex	ρ	1.000	0.176	−0.095	0.331	0.151	0.263	−0.257	−0.280
	p	.	0.098	0.371	0.001	0.156	0.012	0.015	0.007
Age	ρ	0.176	1.000	0.193	0.321	0.811	0.283	−0.115	−0.095
	p	0.098	.	0.069	0.002	0.001	0.007	0.281	0.372
Occup.	ρ	−0.095	0.193	1.000	0.118	0.058	0.064	−0.002	−0.152
	p	0.371	0.069	.	0.267	0.590	0.548	0.981	0.152
EYS	ρ	0.331	0.321	0.118	1.000	0.323	0.771	−0.162	−0.878
	p	0.001	0.002	0.267	.	0.002	0.001	0.127	0.001
TYS	ρ	0.151	0.811	0.058	0.323	1.000	0.304	−0.242	−0.038
	p	0.156	0.001	0.590	0.002	.	0.004	0.022	0.722
MN No	ρ	0.263	0.283	0.064	0.771	0.304	1.000	−0.130	−0.725
	p	0.012	0.007	0.548	0.001	0.004	.	0.223	0.001
Nicotine use	ρ	−0.257	−0.115	−0.002	−0.162	−0.242	−0.130	1.000	0.040
	p	0.015	0.281	0.981	0.127	0.022	0.223	.	0.711
Group exp/cont.	ρ	−0.280	−0.095	−0.152	−0.878	−0.038	−0.725	0.040	1.000
	p	0.007	0.372	0.152	0.001	0.722	0.001	0.711	.
Annual doses	ρ	0.078	0.259	0.273	0.972	0.071	0.364	0.371	−0.753
	p	0.547	0.048	0.019	0.000	0.293	0.000	0.193	0.000

*MN* micronucleus per 1000 binuclear cells, *TYS* total years of service, *EYS* exposed years of service, *Exp* exposed, *Cont* control, *No* number, *Occup* occupartion

**Fig. 1:**
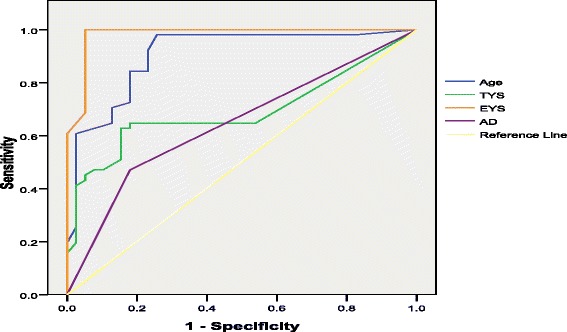
ROC curve for investigated parameters (annual doses, EYS, TYS and age) showing the level to which they explain the occurrence of MN in workers exposed to radiation. Legend: ROC – Receiver Operator Curve – relation of sensitivity and specificity of investigated parameters; TYS – total years of service; EYS – exposed years of service; AD – annual doses

EYS accurately apprehends 86.30 % of MN frequencies, TYS 69.30 %, while age predicts the occurrence MN with the reliability of 67.40 %. Received AD explain correctly the findings of MN in 64.60 % of cases. EYS duration of 14.5 years is the best reference value with a sensitivity 86.4 % and specificity of 80.9 %. TYS duration of 20.5 years is the best reference value with a sensitivity 86.4 % and specificity of 54.4 %. If workers receive more than 1.5 mSy annually (sensitivity 57.1 % and specificity of 83.1 %), they can be expected to have more MN in their cells. Above 43 years of age more MN are produced in the cells, with a sensitivity of 90.9 % and specificity of 47.1 % (Fig. 1).

When all examined parameters were assessed together, using Poisson’s regression, a statistically significant model of their impact on MN formations was constructed (*χ*^2^ = 471.848; *p* = 0.001). Obtained model fits our data reasonably well (*χ*^2^ value/df = 2.838).

$$ \mathsf{M}\mathsf{N}\ \mathsf{p}\mathrm{e}\mathsf{r}\ \mathsf{1}\mathsf{0}\mathsf{0}\mathsf{0}\ \mathsf{bi}\mathrm{n}\mathsf{u}\mathsf{c}\mathsf{l}\mathrm{e}\mathsf{a}\mathsf{r}\ \mathsf{c}\mathrm{e}\mathsf{l}\mathsf{l}\mathsf{s} = \mathsf{1}.\mathsf{231} + \mathsf{0}.\mathsf{244}\times \mathsf{AGE} + \mathsf{0}.\mathsf{301}\times \mathsf{SEX} + \mathsf{0}.\mathsf{017}\times \mathsf{E}\mathsf{Y}\mathsf{S} + \mathsf{0}.\mathsf{683}\times \mathsf{AD} $$

The model shows that older age and female sex of workers, longer duration of exposed years of service and higher annual doses increase the number of MN per 1000 binuclear cells. One EYS year increases the frequency of MN per 1000 binuclear cells 1.017 times. Furthermore, receiving 0.1 mSy raises the frequency of MN per 1000 binuclear cells by 26 %. Specific working places, TYS, workers’ sex and age as well as nicotine use were not significant predictors of formation of MN.

## Discussion

Application of ionizing radiation in many different fields is constantly increasing, including the use for medical purposes. Ionizing radiation is known to induce mutations and cell transformations, predominantly by causing single-strand and double-strand DNA breakage, thereby leading to chromosome instability and carcinogenesis [10]. Of all workers exposed to man-made sources of radiation, medical personnel represent the largest group, but receive relatively low doses. However, some medical uses of radiation, such as nuclear medicine and interventional procedures, may expose personnel to higher doses, and these are subjects of particular concern [11]. Cytogenetic studies report a significant increase of chromosomal damage in exposed subjects, especially interventional cardiologists, operational radiologists and nuclear medicine physicians [12, 13]. On the other hand, some studies concerning mixed population of hospital workers do not report data on cytotoxicity if the measures of prevention and protection from radiation were undertaken adequately [14].

Our study established that the incidence of MN significantly increased for health workers in nuclear medicine departments occupationally exposed to ionizing radiation in comparison to unexposed individuals. Our findings are in accordance with the most other literature data [15–17]. The novelty of this study, that sets it apart from other similar investigations, is construction of a new model for prediction of micronuclei formation in nuclear medicine department workers professionally exposed to ionizing radiation. This model showed that duration of radiation exposure and received annual doses could be used as predictors of MN frequency. Moreover, the originality of our study lies in the fact that it has taken into account the potential impact of working place and specific daily work activities of employees who are in contact with sources of radiation in nuclear medicine department. Additionally, applying the ROC analysis we have, for the first time in this study, set the cut of points of TYS, EYS, AD and age that can imply on higher risk for MN production.

Researchers usually did not find any associations between the MN frequency and the duration of employment except for interventional cardiologists, where MN values are higher in physicians with exposure >10 years in comparison with exposed physicians with hospital work lasting less than 10 years [18]. On the other hand, some authors point out that the length of exposure increases chromosomal aberrations and the number of damaged cells [19]. Moreover, an increased frequency of bi-nucleated and mono-nucleated cells with micronuclei was observed in most of the studies, based on the accumulated radiation dose: statistically significant values were observed above 10 mSv cumulative effective dose along a number of years of employment [19]. Our findings also indicate that the most important predictors for hospital personnel working with radiation sources are received annual doses and duration of EYS. Receiving more than 1.5 mSy annually significantly increases the production and frequency of MN in workers’ cells. Technicians and laboratory workers are at higher risk for MN occurrence as they are generally exposed to more radiation than doctors and engineers.

Evidence in the literature shows a significant correlation of MN frequency with older age and female sex [16, 17]. These findings were confirmed by the results of our study, as well. Women had more MN in the overall population, but among the exposed personal, there were no significant differences regarding MN numbers. Persons occupationally exposed to radiation typically have less than 10 micronuclei if they were exposed for less than 5 years. This all might indicate that radiation itself is more important than workers sex. We also found that, in our population, nuclear medicine department workers aged above 43 years, with more than 20.5 years of TYS and 14.5 years of EYS have more MN. As all received doses were within referent limits, our findings might imply that older personnel should be in some way shielded from adverse effects of radiation more than younger health workers. Perhaps in the age group above 43 years, referent doses should be lowered, which might be the possible methods of preventing adverse effects of radiation in the older population of health workers.

Some studies found that smoking might increase MN numbers in cells of workers exposed to radiation [16]. Still, the majority of literature data agree that it did not affect the MN frequency if examinees consumed less than 20 cigarettes per day, while a significant effect was only registered for heavy smokers [20]. According to our results nicotine use also did not have significant influence on MN formation.

Finally, our results illustrated that low levels of chronic occupational exposure to ionizing radiation causes increased frequency of MN in cells, even when the absorbed dose is below the acceptable limit. Consequently, occupational exposure to radiation can be determined as a risk factor for genotoxicity. The main predictors of the formation and the number of MN in cells, according to our results and the model we constructed, are received annual doses and the length of exposure to ionizing radiation of staff regardless of their specific workplace. Based on the Poisson model, one EYS year increases the frequency of MN per 1000 binuclear cells 1.017 times, while receiving 0.1 mSy raises MN frequency by 26 %. The additional co-factors include age and sex, and they should be closely involved in genetic research monitoring risk assessment of chronic exposure to low doses of radiation of health workers employed in the departments of nuclear medicine. The constructed model might be useful in further research aiming on lowering the referent doses or determining a cut off value for MN in exposed personnel for whom a genotoxic risk is too high. Moreover, it could be used in clinical practice for calculating the number of MN in health workers in case that blood analyses are unavailable, while clinical parameters are known. The equation can be applied to all personnel working with ionizing radiation. It was constructed for our population, but could be used in other countries as well.

MN analysis in human lymphocytes using the cytochalasin B technique has been proposed as a valid and reliable procedure for the assessment of chromosomal damage induced by ionizing radiation. The cytokinesis-blocked micronucleus test has the advantage to detect in interphase both acentric chromosome fragments attributed to DNA breakage and chromosome loss resulting from chromosome lagging in anaphase [21]. Therefore this method was applied in our study as well. This study also demonstrates the usefulness of MN analysis for individual risk assessment in programs of medical surveillance of employees in the departments of nuclear medicine.

This study has some limitations. Although we assessed MN frequency, we did not score nuclear buds and nucleoplasmic bridges. Knowing that one dicentric chromosome manifests as one nucleoplasmic bridge, this additional scoring could provide a stronger causation between IR and potential risk for cancer in occupational settings. However, we are leaving this as well as assessment of other possible genotoxicities for further research. Moreover, currently there were not enough patients to make 5-years or 1-year age groups.

## Conclusions

Parameters such as female gender of workers, older age, higher received annual doses, longer total and exposed years of service increase the number of MN in cells of health workers exposed to radiation. In our population, health workers older than 43 years, with TYS longer than 20.5 years and EYS longer than 14.5 years have significantly more MN. If workers receive more than 1.5 mSy annually, they can be expected to have more MN in their cells. The most important predictors of the micronuclei formation and frequency in nuclear medicine department workers are received annual doses and the length of exposed years of service. Technicians and laboratory workers are at higher risk for occurrence of MN than doctors and engineers, due to receiving higher annual radiation doses.

We developed a model of relationship between clinical parameters and MN numbers showing that both received annual doses and radiation exposure duration could be used as predictors of MN frequency per 1000 binuclear cells. One EYS year increases the frequency of MN per 1000 binuclear cells 1.017 times, while receiving 0.1 mSy raises MN frequency by 26 %.
